# Supplementary material for: Intersecting social and environmental determinants of multidrug-resistant urinary tract infections in East Africa beyond antibiotic use
Source: Nat Commun. 2024 Oct 31;15:9418. doi: 10.1038/s41467-024-53253-x (PMC11528027; doi:10.1038/s41467-024-53253-x)
Supplement: Supplementary file 1 — Supplementary Information [file 41467_2024_53253_MOESM1_ESM.pdf]

Supplementary Materials

Table of Contents

1. Bayesian Profile regression, additional results 2

2. HATUA patient recruitment: sites and healthcare facilities........ 18

3. Missing data and sample characteristics........ 19

4. Measurement of multidrug resistance (MDR). 20

5. Statistical Details- Bayesian Profile Regression 22

6. References 25

1. Bayesian Profile regression, additional results

Figure S1: Description of the 24 clusters: sample size (left)and median MDR risk with CI (right)(n=1610)

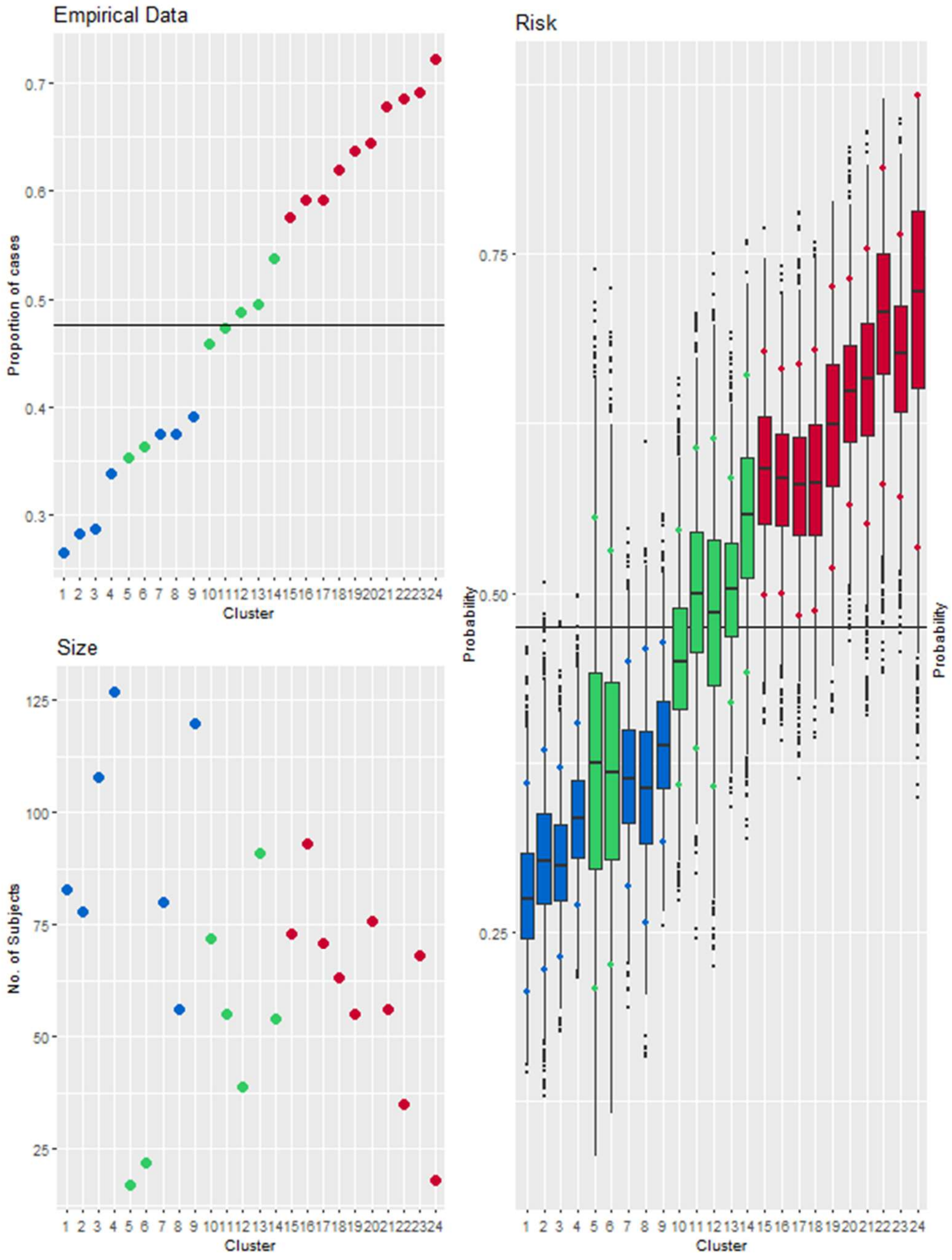

Note: Blue colours represent clusters with low risk of MDR UTI; green colours indicate clusters with average risk of MDR UTI, and red colours indicate clusters with high risk of MDR UTI

.

Figure S2: Difference from the median values for 42 variables in the 7 low-risk MDR UTI clusters.

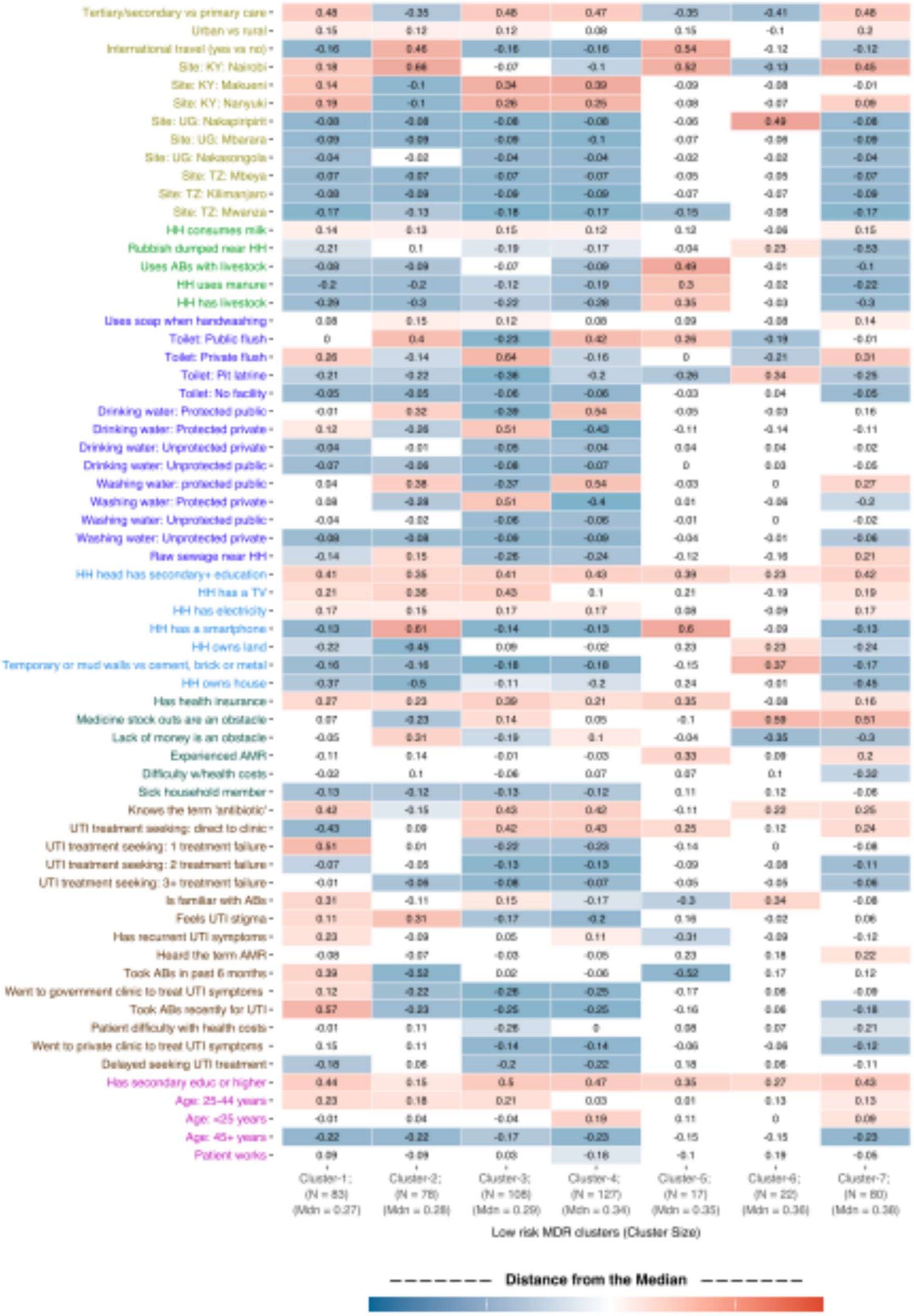

Notes: Figure S2 displays how the 42 important variables (y axis) are distributed within each low-risk MDR cluster (x axis). The variables are grouped thematically and within each theme, ranked according to the strength and direction of the associations with MDR. The numbers in the cells indicate the distance between the proportion of this characteristic in the whole sample and the median probability of having this characteristic in the specific cluster. The shading of the blue and red colours indicates the strength of the prevalence of the factor's category in

the low-risk cluster, with deeper colours showing a higher prevalence. For example, a row which contains majority red blocks indicates that subjects that belong to a low-risk MDR UTI cluster are likely to have this factor characteristic, whereas majority blue blocks indicate that subjects that belong to a low-risk MDR cluster are not likely to have this factor characteristic. A mixture of blue or reds, or more neutral shades indicate no clear signal. Source data are provided as a Source Data file. For more detail, please consult the detailed PReMiuM plots available in Github repository: https://github.com/katykeenan1981/hatuaprofilepaper/blob/main/README.md

Figure S3: Predicted MDR UTI risks for low risk (orange) and high risk (blue) using risk factors from Figure 6, across sites (n=1610). Source data are provided as a Source Data file.

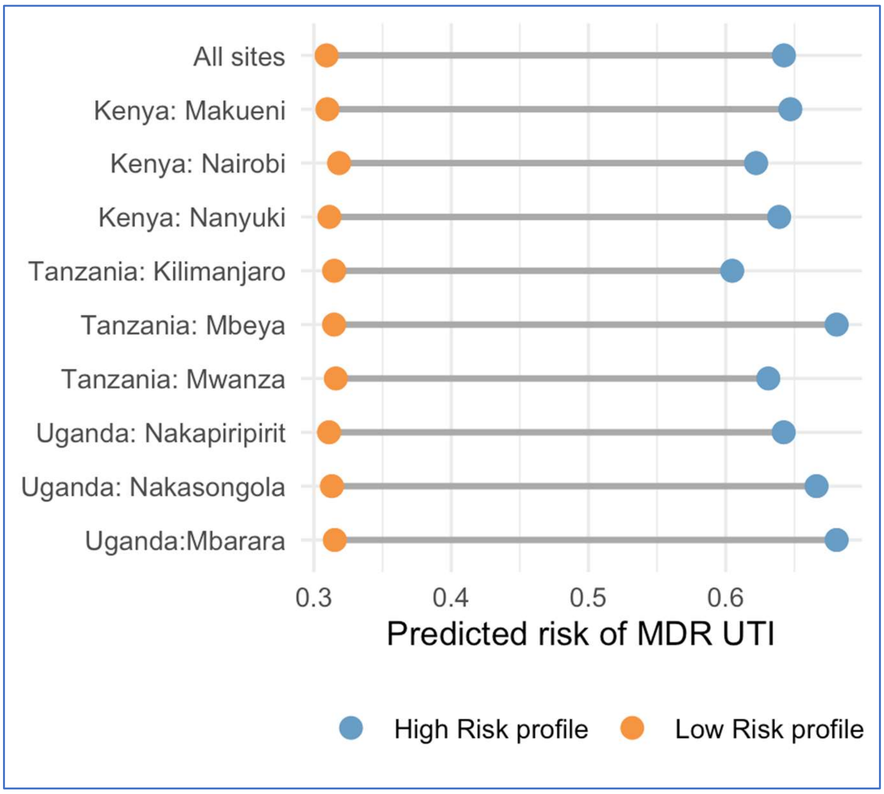

Figure S4: Predicted MDR UTI risks for low risk for very deprived (blue) and not deprived (orange) using HATUA MPI, and across sites (n=1610). Source data are provided as a Source Data file.

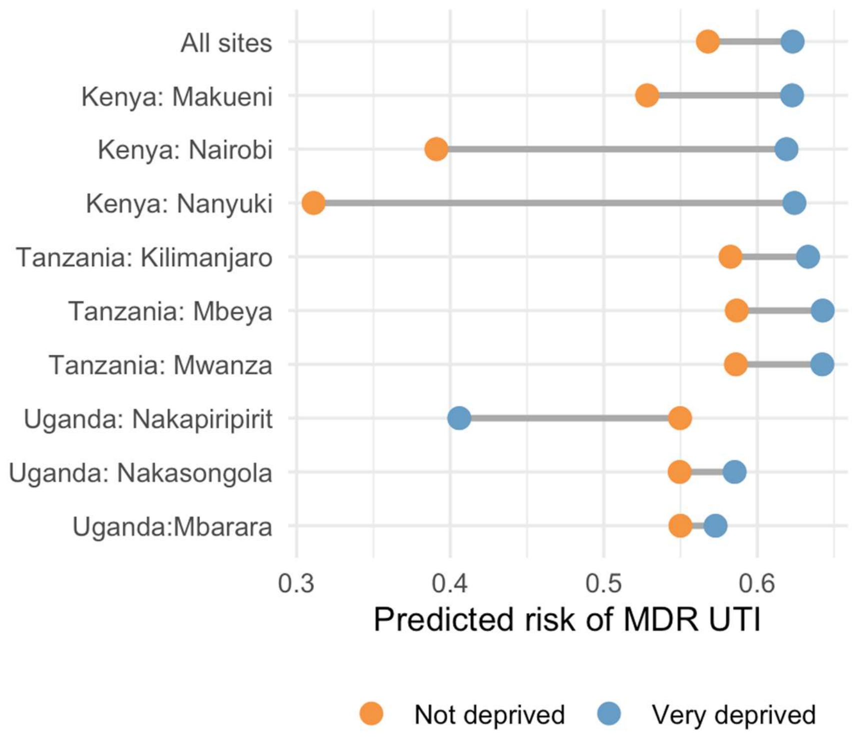

Figure S5: Difference from the median values for 37 variables in the 8 high-risk MDR UTI clusters, women only (n=1,369).

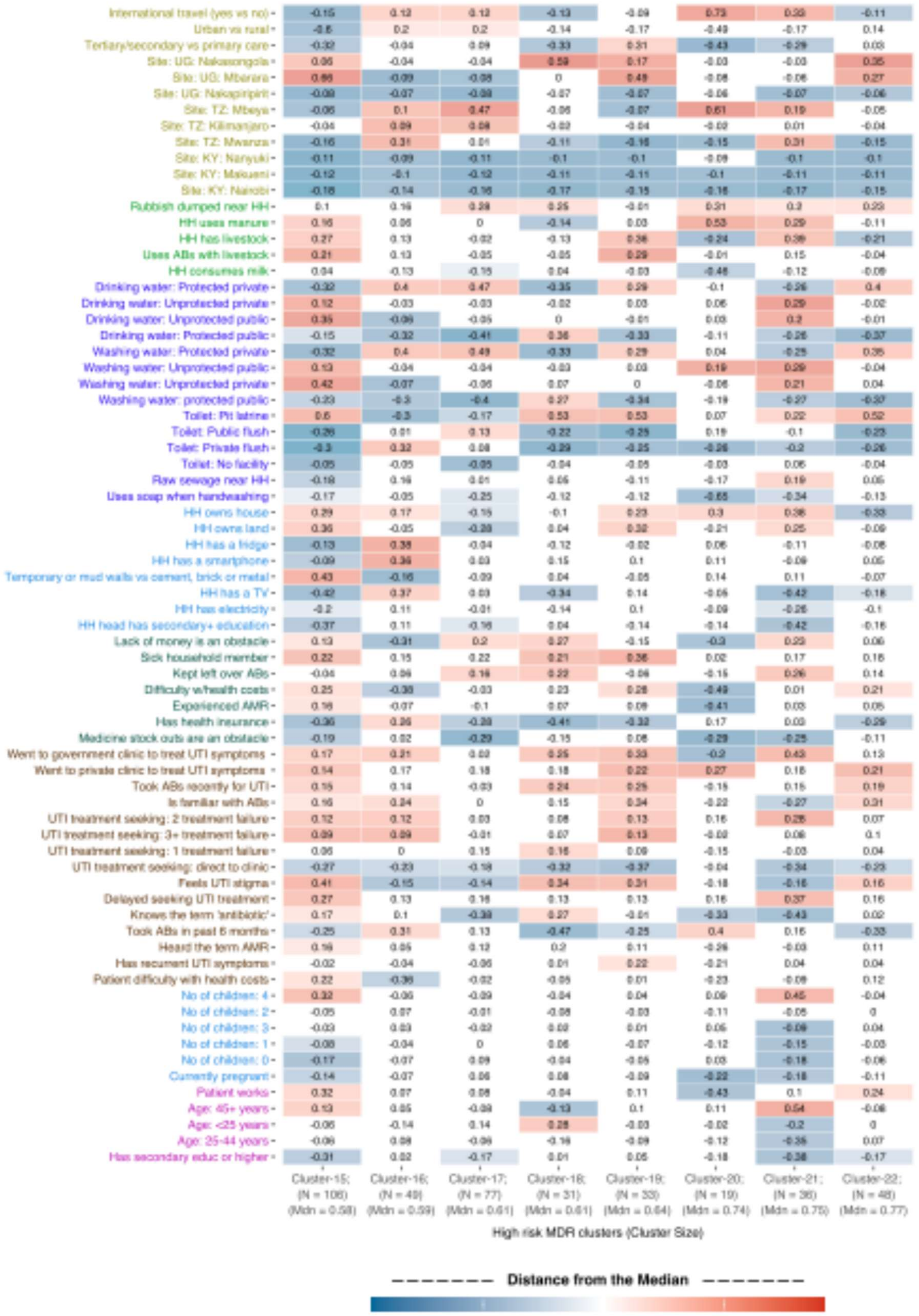

Notes: Figure S5 displays how the 37 important variables (y axis) are distributed within each high-risk MDR cluster (x axis). The variables are grouped thematically and within each theme, ranked according to the strength and direction of the associations with MDR. The numbers in the cells indicate the distance between the proportion of this characteristic in the whole sample and the median probability of having this

characteristic in the specific cluster. The shading of the blue and red colours indicates the strength of the prevalence of the factor's category in the high-risk cluster, with deeper colours showing a higher prevalence. For example, a row which contains majority red blocks indicates that subjects that belong to a high-risk MDR UTI cluster are likely to have this factor characteristic, whereas majority blue blocks indicate that subjects that belong to a high-risk MDR cluster are not likely to have this factor characteristic. A mixture of blue or reds, or more neutral shades indicate no clear signal. Source data are provided as a Source Data file.

Figure S6: Difference from the median values for 37 variables in the 5 low-risk MDR UTI clusters, women only (n=1,369).

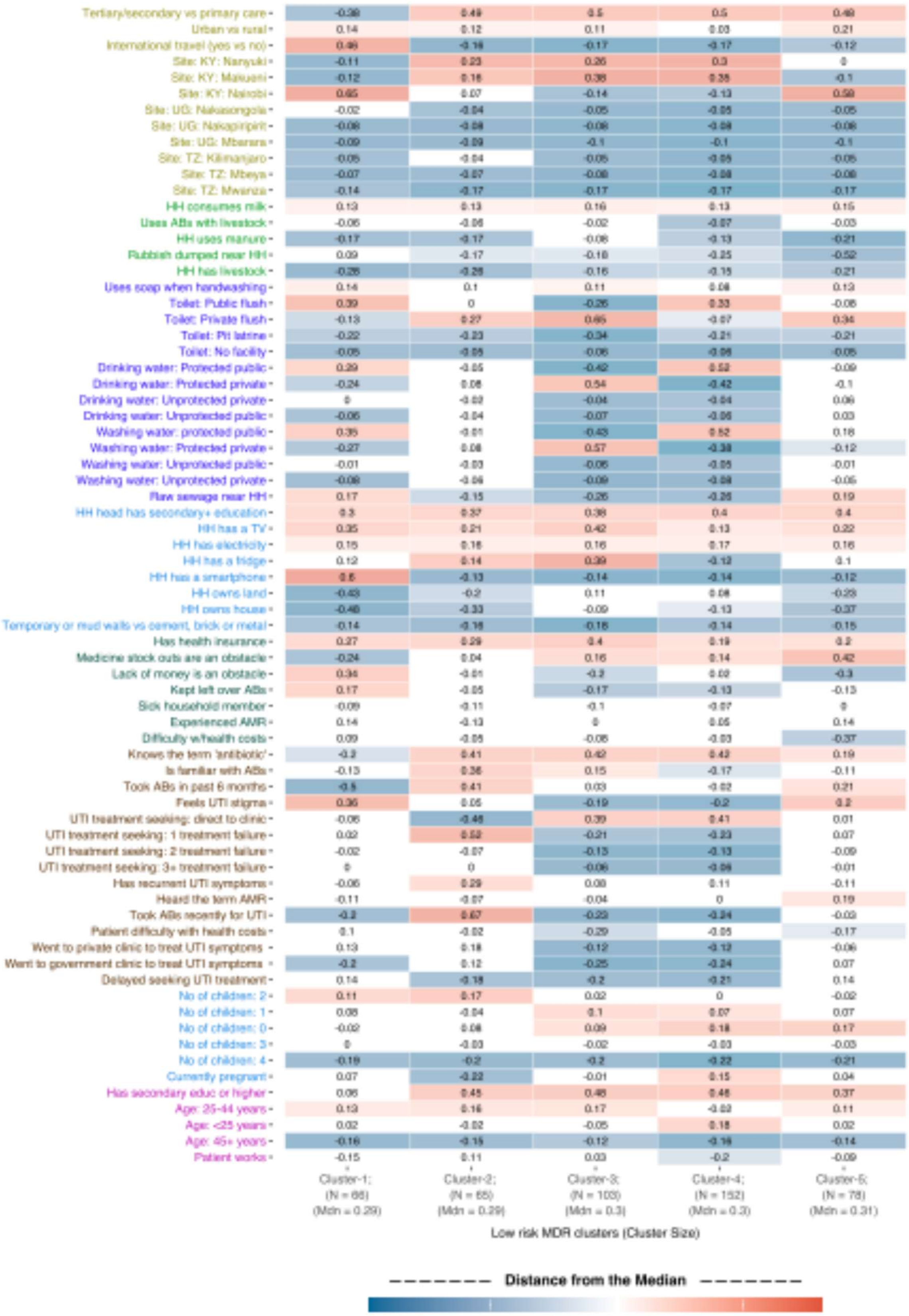

Notes: Figure S6 displays how the 37 important variables (y axis) are distributed within each low-risk MDR cluster (x axis). The variables are grouped thematically and within each theme, ranked according to the strength and direction of the associations with MDR. The numbers in the cells indicate the distance between the proportion of this characteristic in the whole sample and the median probability of having this

characteristic in the specific cluster. The shading of the blue and red colours indicates the strength of the prevalence of the factor's category in the low-risk cluster, with deeper colours showing a higher prevalence. For example, a row which contains majority red blocks indicates that subjects that belong to a low-risk MDR UTI cluster are likely to have this factor characteristic, whereas majority blue blocks indicate that subjects that belong to a low-risk MDR cluster are not likely to have this factor characteristic. A mixture of blue or reds, or more neutral shades indicate no clear signal. Source data are provided as a Source Data file.

Figure S7: Difference from the median values for 39 variables in the 10 high-risk EA ABR clusters.

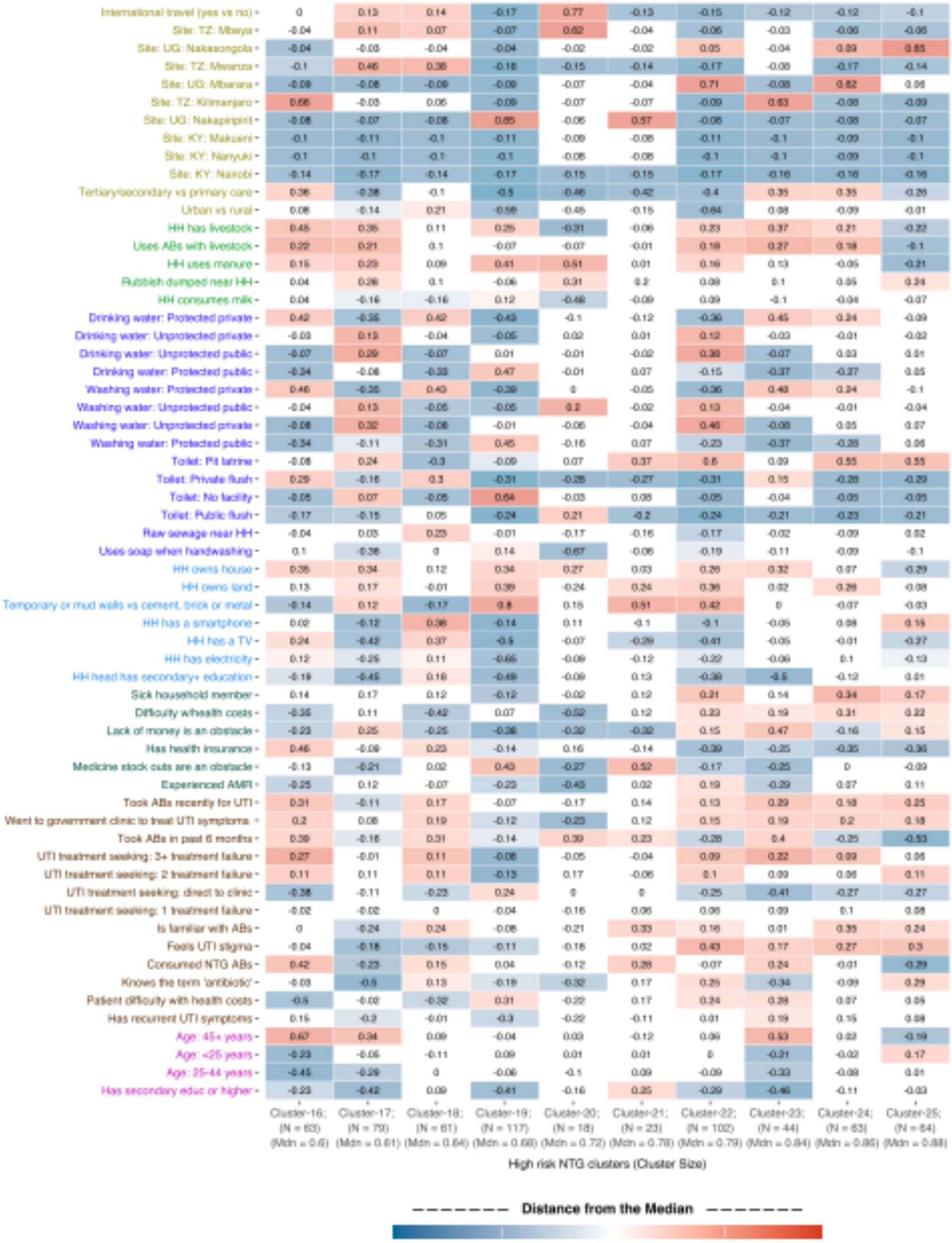

Notes: Figure S7 displays how the 39 important variables (y axis) are distributed within each high-risk MDR cluster (x axis). The variables are grouped thematically and within each theme, ranked according to the strength and direction of the associations with MDR. The numbers in the cells indicate the distance between the proportion of this characteristic in the whole sample and the median probability of having this characteristic in the specific cluster. The shading of the blue and red colours indicates the strength of the prevalence of the factor's category in

the high-risk cluster, with deeper colours showing a higher prevalence. For example, a row which contains majority red blocks indicates that subjects that belong to a high-risk MDR UTI cluster are likely to have this factor characteristic, whereas majority blue blocks indicate that subjects that belong to a high-risk MDR cluster are not likely to have this factor characteristic. A mixture of blue or reds, or more neutral shades indicate no clear signal. Source data are provided as a Source Data file.

Figure S8: Difference from the median values for 39 variables in the 8 low-risk EA ABR clusters.

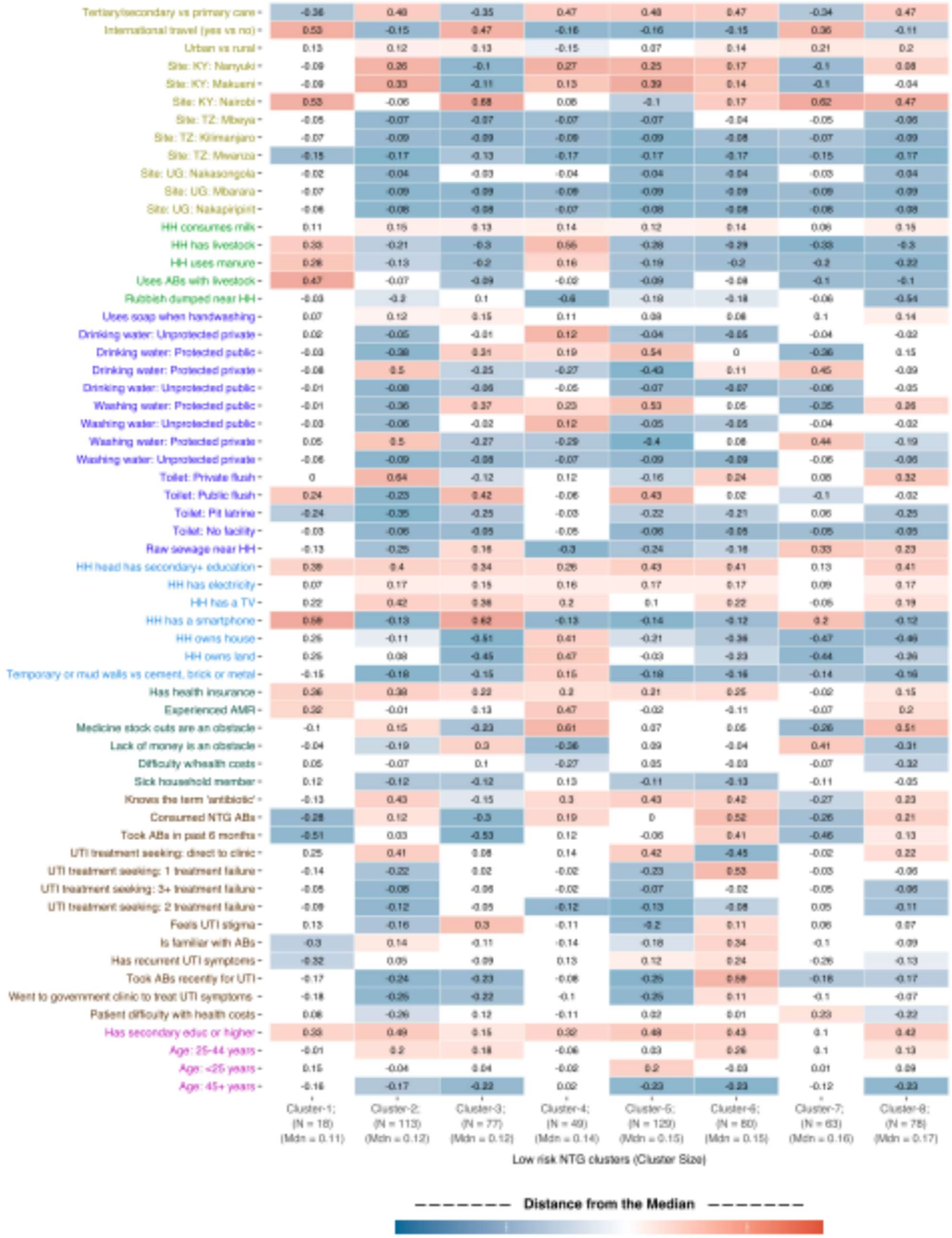

Notes: Figure S8 displays how the 39 important variables (y axis) are distributed within each low-risk MDR cluster (x axis). The variables are grouped thematically and within each theme, ranked according to the strength and direction of the associations with MDR. The numbers in the cells indicate the distance between the proportion of this characteristic in the whole sample and the median probability of having this characteristic in the specific cluster. The shading of the blue and red colours indicates the strength of the prevalence of the factor's category in

the low-risk cluster, with deeper colours showing a higher prevalence. For example, a row which contains majority red blocks indicates that subjects that belong to a low-risk MDR UTI cluster are likely to have this factor characteristic, whereas majority blue blocks indicate that subjects that belong to a low-risk MDR cluster are not likely to have this factor characteristic. A mixture of blue or reds, or more neutral shades indicate no clear signal. Source data are provided as a Source Data file.

Figure S9: Difference from the median values for 41 variables in the 6 high-risk MDR clusters (intermediate as susceptible).

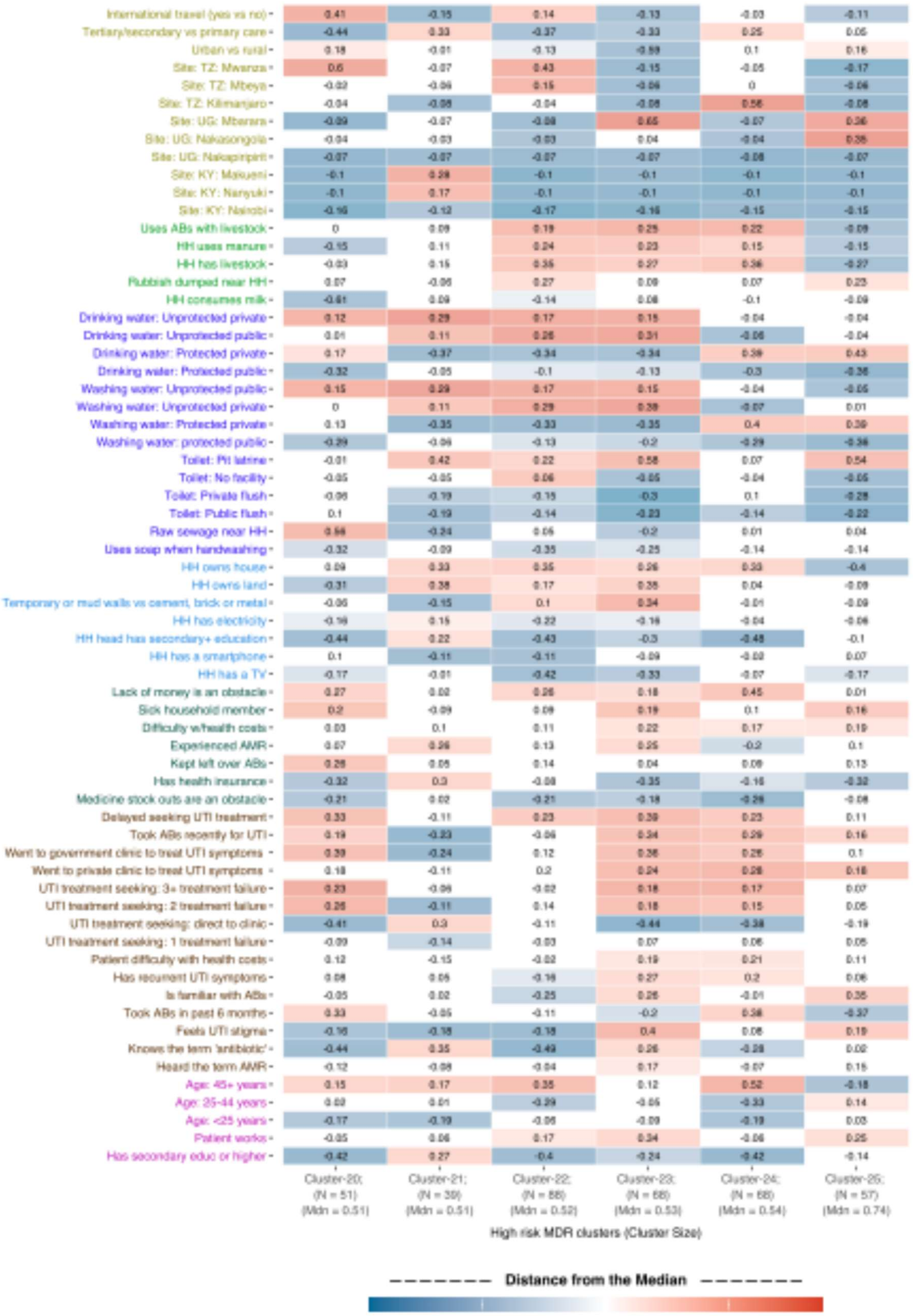

Notes: Figure S9 displays how the 41 important variables (y axis) are distributed within each high-risk MDR cluster (x axis). The variables are grouped thematically and within each theme, ranked according to the strength and direction of the associations with MDR. The numbers in the cells indicate the distance between the proportion of this characteristic in the whole sample and the median probability of having this

characteristic in the specific cluster. The shading of the blue and red colours indicates the strength of the prevalence of the factor's category in the high-risk cluster, with deeper colours showing a higher prevalence. For example, a row which contains majority red blocks indicates that subjects that belong to a high-risk MDR UTI cluster are likely to have this factor characteristic, whereas majority blue blocks indicate that subjects that belong to a high-risk MDR cluster are not likely to have this factor characteristic. A mixture of blue or reds, or more neutral shades indicate no clear signal. Source data are provided as a Source Data file.

Figure S10: Difference from the median values for 41 variables in the 7 low-risk MDR clusters (intermediate as susceptible).

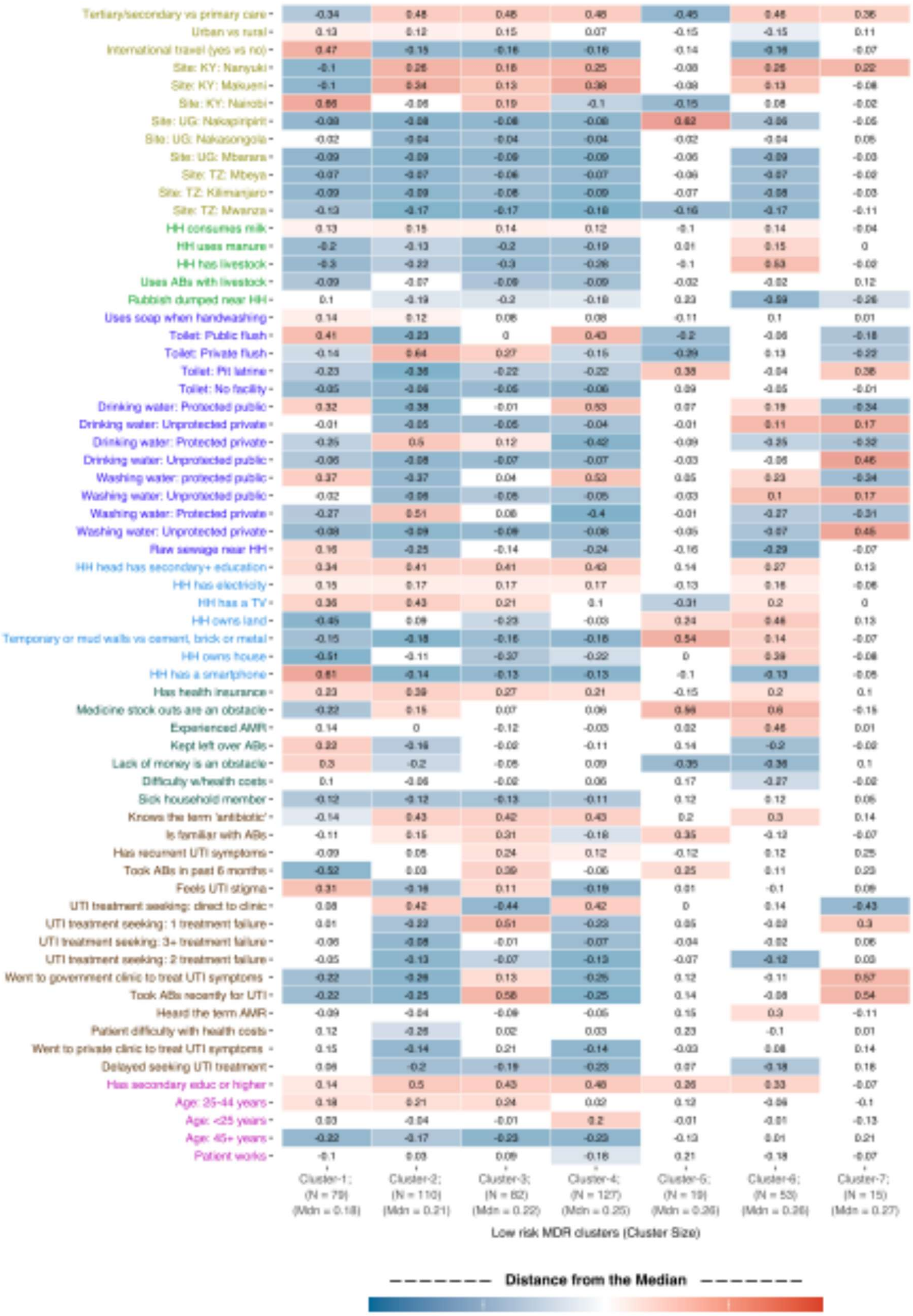

Notes: Figure S10 displays how the 41 important variables (y axis) are distributed within each low-risk MDR cluster (x axis). The variables are grouped thematically and within each theme, ranked according to the strength and direction of the associations with MDR. The numbers in the cells indicate the distance between the proportion of this characteristic in the whole sample and the median probability of having this

characteristic in the specific cluster. The shading of the blue and red colours indicates the strength of the prevalence of the factor's category in the low-risk cluster, with deeper colours showing a higher prevalence. For example, a row which contains majority red blocks indicates that subjects that belong to a low-risk MDR UTI cluster are likely to have this factor characteristic, whereas majority blue blocks indicate that subjects that belong to a low-risk MDR cluster are not likely to have this factor characteristic. A mixture of blue or reds, or more neutral shades indicate no clear signal. Source data are provided as a Source Data file.

2. HATUA patient recruitment: sites and healthcare facilities

HATUA patient recruitment took place in 9 sites- three each in Kenya, Tanzania, and Uganda. Healthcare facilities were chosen across a number of primary, secondary and tertiary care levels. Table S1 presents a breakdown of types of facilities by sites.

Table S1 Patient recruitment sites in Kenya, Tanzania, and Uganda

Country/site	Number of facilities (TOTAL)	Source of funding	Levels recruited from	Recruitment periods
**Kenya**				
Makueni	1	Public	Secondary, tertiary	May-June 2020
Nairobi	4	Public and private	Primary, secondary, tertiary	June 2019-March 2020
Nanyuki	1	Public	Secondary	Feb-May 2020
**Tanzania**				
Kilimanjaro/Moshi	3	Public and private	Primary, secondary, tertiary	August 2019-April 2020
Mbeya	2	Public and private	Primary, secondary, tertiary	July 2019- Aug 2020
Mwanza	5	Public and private	Primary, secondary, tertiary	April 2019-Sept 2020
**Uganda**				
Mbarara	3	Public	Primary, secondary, tertiary	May 2019- July 2020
Nakapiripirit	3	Public	Primary, secondary	January-June 2020
Nakasongola	3	Public and private	Primary, secondary	July 2019-Oct 2019

Levels of facilities in Kenya are identified following the Kenya Health Policy 2014-2013 (http://publications.universalhealth2030.org/uploads/kenya_health_policy_2014_to_2030.pdf, where Primary care incorporates levels 1-3 (Level 1: Community; Level 2: Dispensaries; Level 3: Health centres, secondary care incorporates levels 4 and 5 (Level 4: Primary referral facilities; Level 5; secondary referral facilities) and tertiary care level 6 (tertiary referral facilities).

Levels of facilities in Tanzania were identified using the scheme used in the Tanzanian Health Sector Strategic Plan IV 2015-2020 (https://www.prb.org/wp-content/uploads/2020/06/Tanzania-Health-Sector-Strategic-Plan-IV-2015-2020-1-4.pdf), p.12 Fig 4. where:

Primary care incorporates village level dispensaries, ward level rural health centres, district level hospitals, secondary incorporates regional level (level 2 facilities), and tertiary includes regional or national level referral hospitals (levels 3 and 4 facilities).

Levels of facilities in Uganda are identified following the Health Facility Categories in the National Health Facility Master List 2018 (file:///Users/klk4/Downloads/National%20Health%20Facility%20Master%20List%202018.pdf) where primary care includes health centre II, health centre III, secondary care includes health centre IV, general hospital (levels 4-5) and tertiary care includes referral hospitals, regional referral hospitals, and national referral hospitals (levels 6-8).

3. Missing data and sample characteristics

As shown in Figure 5, HATUA recruited patients in healthcare facilities, processed their microbiological data, then followed up a subset of microbiologically confirmed UTI patients to their households. In Table S2 we show the sociodemographic, health and location characteristics of the patient sample at three stages: patient recruited with microbiologically confirmed UTI, the subset of those with valid AST data, and the final analysis subset of those who were successfully followed to the household. When trying to understand possible bias from missing data the best comparison is between columns A and C. When we do this, we do not observe large differences in age distribution, sex, working status, education, or health status. In the analysis sample, there are also proportionally more patients from Kenyan sites (especially Nairobi) and fewer from both Tanzanian (particularly Mwanza).

Table S2. Characteristics of patients in the analysis sample (column C) compared to all those who were recruited in the study

	A	B	C (analysis sample)
	Of those, with microbiologically confirmed UTI	Of those, with valid AST/MDR data	Of those with complete household follow-up
	N =2332	N=2063	N=1610
**Age**			
Under 25 years	27.4	27.5	26.1
25-44 years	48.5	49.1	48.8
45 and over	24.0	23.4	24.7
Missing	0.1	0.1	0.4
**Sex**			
Male	14.9	14.3	15.0
Female	85.1	85.7	85.0
**Working Status**			
Formal employment	18.7	18.1	17.2
Informal employment	40.3	41.6	42.5
Homemaker	27.7	27.3	27.0
Not working	12.9	12.6	12.9
Missing	0.5	0.4	0.4
**Highest education**			
No qualifications	15.1	14.6	15.6
Primary	35.3	34.4	35.3
Secondary	33.1	33.7	32.4
Higher than secondary	16.4	17.3	16.7
**Health status**			
No pre-existing conditions	86.6	86.8	85.0
Has long-term NCD[1] or HIV	13.4	13.2	15.0
**Region**			
Kenya: Makueni	10.7	11.8	11.4
Kenya: Nairobi	15.7	16.5	17.7
Kenya: Nanyuki	12.7	13.9	11.1
Tanzania: Kilimanjaro	10.2	8.8	9.8
Tanzania: Mbeya	7.0	7.0	8.0
Tanzania: Mwanza	22.6	20.4	18.3
Uganda: Mbarara	8.3	8.6	10.1
Uganda: Nakapiripirit	8.5	8.3	8.6
Uganda: Nakasongola	4.3	4.6	5.0

[1] Includes heart conditions, cancer, mental illness, gastrointestinal problems, allergies, asthma or bone problems

4. Measurement of multidrug resistance (MDR).

Table S3. ABs considered for MDR calculations.

Gram negative	Amoxicillin/ clavulanate (AMC)	Ampicillin (AMP)	Ceftazidime/ Ceftriaxone (CAZ/CRO)	Ciprofloxacin (CIP)	Gentamicin (GEN)	Nitrofurantoin (NIT)	Trimethoprim (TMP)		
Escherichia coli	AMC	AMP	CAZ/CRO	CIP	GEN	NIT	TMP		
Shigella spp.	AMC	AMP	CAZ/CRO	CIP	GEN	NIT	TMP		
Proteus spp.	AMC	AMP	CAZ/CRO	CIP	GEN	NIT	TMP		
Salmonella spp.	AMC	AMP	CAZ/CRO	CIP	GEN	NIT	TMP		
Serratia spp.	-	-	CAZ/CRO	CIP	GEN	NIT	TMP		
Klebsiella spp.	AMC	-	CAZ/CRO	CIP	GEN	NIT	TMP		
Citrobacter spp.	-	-	CAZ/CRO	CIP	GEN	NIT	TMP		
Enterobacter spp.	-	-	CAZ/CRO	CIP	GEN	NIT	TMP		
Morganella spp.	-	-	CAZ/CRO	CIP	GEN	NIT	TMP		
Pantoea spp.	-	-	CAZ/CRO	CIP	GEN	NIT	TMP		
Providencia spp.	-	-	CAZ/CRO	CIP	GEN	NIT	TMP		
Acinetobacter spp.	-	-	CAZ/CRO	CIP	GEN	-	TMP		
Pseudomonas spp.	-	-	CAZ	CIP	GEN	-	-		
Gram positive	Cefoxitin (FOX)	Erythromycin (ERY)	Linezolid (LNZ)	Ciprofloxacin (CIP)	Gentamicin (GEN)	Nitrofurantoin (NIT)	Trimethoprim (TMP)	Tetracycline (TCY)	Vancomycin (VAN)
Staphylococcus spp.	FOX	ERY	-	CIP	GEN	NIT	TMP	TCY	-
Enterococcus spp.	-	ERY	LNZ	CIP	-	NIT	-	TCY	VAN
Streptococcus spp.	-	ERY	LNZ	-	-	NIT	-	TCY	VAN

Table S4. List of ABs recommended for use for treating uncomplicated upper and lower UTI according to country National Treatment Guidelines (NTG)

	**Kenya NTG**[1] (adult outpatients- not admitted to hospital)	**Tanzania NTG**[2] (adult outpatients- not admitted to hospital)	**Uganda NTG**[3] (adult outpatients- not admitted to hospital)
Amoxicillin	a, b		c
Amoxicillin/ Clavulanic Acid		b, c	
Ampicillin	a		b, c
Ceftriaxone			b, c
Ciprofloxacin		a, b	a, b
Cotrimoxozole/trimethoprim	a, b		
Gentamicin			b, c
Nitrofurantoin	a		a, c

a= uncomplicated lower UTI

b= uncomplicated upper UTI (includes for Uganda, first and second line options)

c= treatments recommended for pregnant women only

1 http://guidelines.health.go.ke:8000/media/Clinical_Guidelines_Vol_II_Final.pdf

2 https://hssrc.tamisemi.go.tz/storage/app/uploads/public/5ab/e9b/b21/5abe9bb216267130384889.pdf

3 https://www.prb.org/wp-content/uploads/2018/05/Uganda-Clinical-Guidelines-2016-National-Guidelines-for-Management-of-Common-Conditions.pdf

For Kenyan patients, we created a binary variable measuring if the patient was resistant to any of the following: ampicillin, cotrimoxazole/trimethoprim or nitrofurantoin. In Tanzanian patients, we created a binary variable indicating resistance to either amoxicillin/clavulanic acid or ciprofloxacin. Among Ugandan patients, we created a binary variable measuring resistance to either ampicillin, ceftriaxone, ciprofloxacin, gentamicin or nitrofurantoin. .

5. Statistical Details- Bayesian Profile Regression

We use Bayesian profile regression [1,2] to cluster subjects or patients based on covariates collected for each patient and analyse different risk exposures to the response variable of ABR for different groups of patients identified. The R package PReMiuM [3] is employed to fit the model.

We first describe the clustering model underneath profile regression. In our analysis, all clustering variables are categorical, and each follows a categorical distribution. Suppose there are n patients and J variables used for clustering. For cluster c, denote by $\Phi_j^c = (\Phi_{j,1}^c, \dots, \Phi_{j,w_j}^c)$ the cluster-specific probabilities that variable j takes a value from 1 to w_j, where w_j denotes the total number of categories for variable j. Further denote $\Phi^c = (\Phi_1^c, \dots, \Phi_J^c)$ and $\mathbf{\Phi} = (\Phi^1, \Phi^2, \dots)$. The likelihood can be written as follows:

$$P(X_i|Z_i = c, \mathbf{\Phi}) = \prod_{j=1}^{J} P(X_{ij}|Z_i = c, \mathbf{\Phi}) \qquad (\mathbf{1})$$

$$= \prod_{j=1}^{J} \Phi_j^c(X_{ij}),$$

where $X_i = (X_{i1,}X_{i2,}\dots X_{iJ,})$ denotes a vector of J observed variables for patient i. Z_i represents the cluster allocation variable and $\boldsymbol{Z} = (Z_{1,}\, Z_{2,}\dots Z_n)$. Denote by $\boldsymbol{\psi} = (\psi_{1,}\, \psi_{2,}\dots)$ the vector of mixture weights such that $\sum_c \psi_c = 1$ and,

$$P(Z_i = c|\boldsymbol{\psi}) = \psi_{\boldsymbol{c}} \qquad (\mathbf{2})$$

Bayesian profile regression uses the Dirichlet process mixture model (DPMM) to perform the clustering. Denote by $DP\ (\alpha, G_0)$ the Dirichlet process with concentration parameter α and base distribution G_0. The stick-breaking representation of the DPMM given by Papaspiliopoulos and Roberts[4] comes from the stick-breaking representation of the DP[5], where the DPMM can be viewed as an infinite mixture model, namely

$$X_i\ |\boldsymbol{Z}, \mathbf{\Phi} \sim \prod_{j=1}^{J} Cat\ (X_{ij}\ |\ \Phi_j^{Z_i}),$$

$$Z_i\ |\boldsymbol{\psi} \sim \prod_{c}^{\infty} \psi_c\, \delta_c,$$

$$\psi_c = V_c \sum_{l<c} (1 - V_l), \psi_1 = V_1, \qquad (3)$$

$$V_c \sim Beta\ (1, \alpha),$$

$$\Phi^c\ |\ G_0 \sim G_0, c = 1, 2, 3, \dots$$

where Cat represents the categorical distribution; $Beta$ represents the Beta distribution and δ_c is the Dirac delta function centered at c. Note that setting the number of components in the DPMM to be infinite is a mathematical way to express uncertainty over the number of components. The best

representative clustering for the patients is obtained by the post-processing procedure detailed in Molitor et al[1]. In this manuscript, it is explained how uncertainty is incorporated in our inferences by considering the samples from all Markov Chain Monte Carlo (MCMC) iterations obtained during the Bayesian inferential sampling procedure.

Since the concentration parameter α can affect the number of clusters identified by the DPMM, a relatively vague prior is placed on α.

To model the risk for a positive outcome associated with the different patient groups, a regression model is added to the overall modelling framework. In the context of our analysis, the response variable is a binary variable describing the presence of MDR. A logistic regression is used, namely

$$logit(\pi_i) = \theta_{Z_i} + \beta \boldsymbol{f}_i, \qquad (4)$$

where π_i denotes the probability of subject i having MDR. $\boldsymbol{f}_i = (f_i^1, f_i^2 \ldots, \ldots, f_i^P)$ is a vector of P *optional* confounding or adjusting variables one may elect to include in the regression model. For this manuscript, preliminary analyses showed that inferences were clearer and more interpretable when all covariates were included as clustering variables. θ_{Z_i} are cluster specific risk effect parameters.

The regression model in (4) is fitted simultaneously with the DPMM in (3). Therefore, the response variable also exerts influence on the cluster allocation of patients.

For each cluster of patients, the baseline risk of having MDR can be calculated as

$$p_{z_i} = \frac{exp\,(\theta_{Z_i})}{1 + exp(\theta_{Z_i})}$$

Under the Bayesian framework, the posterior distribution of θ_{Z_i} can be obtained, which enables us to make inferences for the baseline risk of each cluster. In our analysis, we calculate the 95% credible interval of $p_{z_i}$and compare it to the overall sample proportion of patients having MDR, denoted as π. If the lower bound of the credible interval is above π, we regard the corresponding cluster of patients as having high risk of getting MDR. If the upper bound of the credible interval is below π, we regard the corresponding cluster of patients as having low risk of getting MDR. If π is contained in the credible interval, we regard the corresponding cluster of patients as having average risk of getting MDR.

Due to the relatively large number of covariates included in the clustering model, it is of interest to determine which covariates are important for forming the clusters. A variable selection procedure is considered. We utilise the variable selection formulation that involves cluster specific indicators, as proposed by Papathomas et al[2].This results in estimating continuous selection variables $\rho = (\rho_1, \ldots, \ldots \rho_J)$. Note that $\rho_j \in [0,1]$ for $j = \ldots 2, \ldots, J$. The closer ρ_j is to 1 the corresponding covariate j is deemed to be important for determining the overall clustering structure. In contrast, the closer ρ_j is to 0, the corresponding covariate j is considered to be irrelevant for forming clusters. So, the ρ_j can be viewed as variable selection probabilities, where variable j is deemed important when the posterior mean or median of $\rho_j$exceeds some predefined threshold.

A covariate profile for each cluster of patients can be obtained. More specifically, the 95% credible interval of $\Phi_{j,q}^{c}$ is calculated for variables that are deemed important based on the posterior inferences for the variables in ρ. The credible interval for each $\Phi_{j,q}^{c}$is compared to $p_{j,p}$, where $p_{j,q}$ is the overall sample proportion for variable j when it equals category q. If the lower bound of the credible interval is above $p_{j,p}$. we regard the corresponding cluster of patients as having high probability of having category q for variable j. In the plots produced by PReMiuM (see Figures S3), these CIs are shown with red colour. If the upper bound of the credible interval is below $p_{j,p}$, we regard the corresponding cluster of patients as having low probability of having category q for variable p. In the plots produced by PReMiuM, these CIs are shown with blue colour. To ensure convergence of the MCMC sampling scheme we obtained 5000 samples after a burn-in of 800,000 iterations. Convergence checks showed no reason for concern, with the best representative clustering after different MCMC starting points matching by more than 98%. (RAND index greater than 0.98; see [6].

As a follow-up step, to aid interpretation and translate the findings, we calculated predictive profiles using PReMiuM software, according to two different methods. First, we used a priori definitions of multidimensional poverty risk based the profiles on patients being not/very deprived according to the HATUA multidimensional poverty index (MPI) considering education and standard of living domains derived in our earlier paper on misuse [7].

Not deprived profile:

patient and household head - at least secondary education
Assets; has electricity, TV, motorised vehicle, computer, fridge, smartphone, flush toilet
House walls made of brick or concrete.

Deprived profile:

patient and household head -less than secondary education,
Assets: does not have electricity, TV, motorised vehicle, computer, fridge, smartphone, or flush toilet, house walls not made of brick or concrete.

We also computed predictive profiles based on the profile regression results, using the variables/characteristics we identified as having clear signals for high and low risk MDR clusters listed in Fig 6. Those were:

High risk: age 45+, less than secondary education, urban residence, no electricity, owns own house, no health insurance, does not use soap when handwashing, rubbish dumping, contact with manure, keeps livestock, uses ABs to raise livestock, treatment delay, 3+ treatment failures, went to government clinic, familiar with at least 4 types of ABs, has a sick household member, no obstacle to getting medication.

Low risk: age <45 years, at least secondary education, rural residence, recruited in secondary/tertiary healthcare, has electricity, has health insurance, uses soap when handwashing, no rubbish dumping, does not use ABs to raise livestock, no treatment delay, went straight to clinic (no treatment failures), knows the term ‘antibiotic’, no sick household members, reports obstacle to getting medication.

6. References

1 Molitor J, Papathomas M, Jerrett M, Richardson S. Bayesian profile regression with an application to the National survey of children's health. *Biostatistics* 2010; **11**: 484–98.

2 Papathomas M, Molitor J, Hoggart C, Hastie D, Richardson S. Exploring Data From Genetic Association Studies Using Bayesian Variable Selection and the Dirichlet Process: Application to Searching for Gene × Gene Patterns. *Genetic Epidemiology* 2012; **36**: 663–74.

3 Liverani S, Hastie DI, Azizi L, Papathomas M, Richardson S. PReMiuM: An R Package for Profile Regression Mixture Models Using Dirichlet Processes. *J Stat Softw* 2015; **64**: 1–30.

4 Papaspiliopoulos O, Roberts GO. Retrospective Markov chain Monte Carlo methods for Dirichlet process hierarchical models. *Biometrika* 2008; **95**: 169–86.

5 Sethuraman J. A Constructive Definition of Dirichlet Priors. *Statistica Sinica* 1994; **4**: 639–50.

6 Milligan GW, Cooper MC. A Study of the Comparability of External Criteria for Hierarchical Cluster Analysis. *Multivariate Behavioral Research* 1986; **21**: 441–58.

7 Green DL, Keenan K, Fredricks KJ, *et al.* The role of multidimensional poverty in antibiotic misuse: a mixed-methods study of self-medication and non-adherence in Kenya, Tanzania, and Uganda. *The Lancet Global Health* 2023; **11**: e59–68.
