# Supplementary material for: Intersecting social and environmental determinants of multidrug-resistant urinary tract infections in East Africa beyond antibiotic use
Source: Nat Commun. 2024 Oct 31;15:9418. doi: 10.1038/s41467-024-53253-x (PMC11528027; doi:10.1038/s41467-024-53253-x)
Supplement: Supplementary file 2 — Reporting Summary [file 41467_2024_53253_MOESM2_ESM.pdf]

nature portfolio

Corresponding author(s): Katherine Keenan

Last updated by author(s): Sep 16, 2024

Reporting Summary

Nature Portfolio wishes to improve the reproducibility of the work that we publish. This form provides structure for consistency and transparency in reporting. For further information on Nature Portfolio policies, see our Editorial Policies and the Editorial Policy Checklist.

Statistics

For all statistical analyses, confirm that the following items are present in the figure legend, table legend, main text, or Methods section.

n/a	Confirmed	
☐	☒	The exact sample size (*n*) for each experimental group/condition, given as a discrete number and unit of measurement
☐	☒	A statement on whether measurements were taken from distinct samples or whether the same sample was measured repeatedly
☐	☒	The statistical test(s) used AND whether they are one- or two-sided<br>*Only common tests should be described solely by name; describe more complex techniques in the Methods section.*
☐	☒	A description of all covariates tested
☐	☒	A description of any assumptions or corrections, such as tests of normality and adjustment for multiple comparisons
☐	☒	A full description of the statistical parameters including central tendency (e.g. means) or other basic estimates (e.g. regression coefficient) AND variation (e.g. standard deviation) or associated estimates of uncertainty (e.g. confidence intervals)
☐	☒	For null hypothesis testing, the test statistic (e.g. *F*, *t*, *r*) with confidence intervals, effect sizes, degrees of freedom and *P* value noted<br>*Give P values as exact values whenever suitable.*
☐	☒	For Bayesian analysis, information on the choice of priors and Markov chain Monte Carlo settings
☒	☐	For hierarchical and complex designs, identification of the appropriate level for tests and full reporting of outcomes
☒	☐	Estimates of effect sizes (e.g. Cohen's *d*, Pearson's *r*), indicating how they were calculated

Our web collection on statistics for biologists contains articles on many of the points above.

Software and code

Policy information about availability of computer code

Data collection	This is detailed in the protocol (cited in the manuscript)
Data analysis	We use R and R Studio version 4.3.2. The Bayesian profile regression was performed using PReMiuM package version 3.2.13. All code is deposited in github: https://github.com/katykeenan1981/hatuaprofilepaper

For manuscripts utilizing custom algorithms or software that are central to the research but not yet described in published literature, software must be made available to editors and reviewers. We strongly encourage code deposition in a community repository (e.g. GitHub). See the Nature Portfolio guidelines for submitting code & software for further information.

Data

Policy information about availability of data

All manuscripts must include a data availability statement. This statement should provide the following information, where applicable:

- Accession codes, unique identifiers, or web links for publicly available datasets
- A description of any restrictions on data availability
- For clinical datasets or third party data, please ensure that the statement adheres to our policy

The data that support the findings of this study is available according to data sharing policy of the partners in the three participating countries, which restricts access due to ethical issues. The data forms part of a larger linked dataset, with ongoing analysis. To request access, please contact the Principal Investigator of the

HATUA Consortium Professor Matthew Holden (mtgh@st-andrews.ac.uk) or the corresponding author (katherine.keenan@st-andrews.ac.uk). At the time of publication, further reuse of the data for analysis would require collaboration in the ongoing work of the HATUA Consortium.

Research involving human participants, their data, or biological material

Policy information about studies with human participants or human data. See also policy information about sex, gender (identity/presentation), and sexual orientation and race, ethnicity and racism.

Reporting on sex and gender	Included
Reporting on race, ethnicity, or other socially relevant groupings	Included
Population characteristics	Included
Recruitment	Included
Ethics oversight	Included

Note that full information on the approval of the study protocol must also be provided in the manuscript.

Field-specific reporting

Please select the one below that is the best fit for your research. If you are not sure, read the appropriate sections before making your selection.

☐ Life sciences ☒ Behavioural & social sciences ☐ Ecological, evolutionary & environmental sciences

For a reference copy of the document with all sections, see nature.com/documents/nr-reporting-summary-flat.pdf

Behavioural & social sciences study design

All studies must disclose on these points even when the disclosure is negative.

Study description	Quantitative cross-sectional data. This study design was chosen to explore the inter-relationships between a wide number of variables at a single point in time. Longitudinal studies would have been challenging given the cost and opportunity.
Research sample	1610 adult outpatients (aged 18 years and older, or those 14-18 years and pregnant, who comprised 1% of the sample) were recruited from several healthcare facilities in three countries (Kenya: Makueni, Nairobi, Nanyuki; Tanzania: Kilimanjaro, Mbeya and Mwanza, Uganda: Mbarara, Nakapiripirit, and Nakasongola). The outpatients presented to the doctor with urinary tract infection symptoms. During face-to-face consultations, doctors or clinical officers identified patients with symptoms indicative of UTI for inclusion to the study. There was no blinding: researchers and participants were aware of the topic of study (hypotheses n/a in such an exploratory study).
Sampling strategy	As per the protocol, sites were chosen to represent different levels of urbanisation, socioeconomic status and environmental exposures. Healthcare facilities were predominantly government-funded and included both primary, secondary, and tertiary levels of care in all countries (see Table S3 for details and recruitment dates). COVID-19 pandemic restrictions affected recruitment, having a higher impact in Kenya, where sampling took place over a shorter period and in fewer (higher level) facilities. In two Kenyan sites (Makueni and Nanyuki) we sampled only from secondary/tertiary hospitals, so proportionally fewer patients in Kenya are drawn from primary care . Each country had a minimum target sample size of 600 patients with microbiologically confirmed UTI (sample size calculations are reported in the study protocol).
Data collection	When selected for inclusion at the clinic, after consenting, patients provided a mid-stream urine sample (this was collected by the patient themselves) and (helped by a trained social science fieldworker) answered a questionnaire on treatment-seeking, AB use, health factors, knowledge and attitudes around ABs, and socio-demographic characteristics (n=6,804). Among patients with microbiologically confirmed UTI (defined by the presence of >104 colony-forming units per millilitre (CFU/mL) of one or two uropathogens) and who consented to be recontacted, we conducted follow-up interviews in person in their homestead about a month later (mean days 31, IQR 5-42). At the household, a questionnaire was administered to the patient, or another adult member of their household, which covered household composition, socioeconomic factors, sanitation and hygiene, illness and health-seeking behaviour, and livestock practices (n=1,610). Interviewers also made observations of environmental features: sanitation, livestock, and hygiene practices.
Timing	Between February 2019 and September 2020
Data exclusions	Figure 5 shows the recruitment flow. To reach out analysis sample, we recruited 6827 symptomatic patients. Of these, we excluded 4474 who were UTI negative. Then we discarded a further 263 who did not have adequate data to determine multi-drug resistance. Then, we excluded a further 453 who did not have linked household follow-up data.
Non-participation	At the clinic, less than 1% declined to participate, and this did not vary by site. Among those who were followed to the household, a further 360 (17.4% of the sample) either refused or could not be contacted.

Randomization	There was no group allocation. This was an observational cross-sectional study where covariates were measured (many, including demographic, economic and social factors, and area-level covariates). They were not controlled but form part of the cluster allocation - and thus are taken account of in the Bayesian profile regression.

Reporting for specific materials, systems and methods

We require information from authors about some types of materials, experimental systems and methods used in many studies. Here, indicate whether each material, system or method listed is relevant to your study. If you are not sure if a list item applies to your research, read the appropriate section before selecting a response.

Materials & experimental systems

n/a	Involved in the study
☒	☐ Antibodies
☒	☐ Eukaryotic cell lines
☒	☐ Palaeontology and archaeology
☒	☐ Animals and other organisms
☐	☒ Clinical data
☒	☐ Dual use research of concern
☒	☐ Plants

Methods

n/a	Involved in the study
☒	☐ ChIP-seq
☒	☐ Flow cytometry
☒	☐ MRI-based neuroimaging

Clinical data

Policy information about clinical studies

All manuscripts should comply with the ICMJE guidelines for publication of clinical research and a completed CONSORT checklist must be included with all submissions.

Clinical trial registration	n/a
Study protocol	*Note where the full trial protocol can be accessed OR if not available, explain why.*
Data collection	Several healthcare facilities in three countries (Kenya: Makueni, Nairobi, Nanyuki; Tanzania: Kilimanjaro, Mbeya and Mwanza, Uganda: Mbarara, Nakapiripirit, and Nakasongola). Between February 2019 and September 2020
Outcomes	Our primary outcome was multidrug-resistant urinary tract infection (MDR UTI) (a binary classification) defined as urinary isolates resistant to at least one agent in three or more defined categories of antimicrobial agents, following the European Centre for Disease Prevention and Control (ECDC) guidelines.We modified this to include nitrofurantoin and trimethoprim, two ABs routinely used for treating UTIs in this region that are not included in the ECDC guidelines (Supplementary Table S5). In addition, for those species/genera not incorporated in the ECDC, i.e. Salmonella spp., Shigella spp., and Streptococcus spp., the MDR rates were calculated as above, but considering the resistance to a selected pool of tested ABs (Table S5). For the main analysis, isolates that showed intermediate resistance to a given AB were considered resistant, following local clinical practice.

Plants

Seed stocks	n/a
Novel plant genotypes	n/a
Authentication	n/a
