# Supplementary material for: Intersecting social and environmental determinants of multidrug-resistant urinary tract infections in East Africa beyond antibiotic use
Source: Nat Commun. 2024 Oct 31;15:9418. doi: 10.1038/s41467-024-53253-x (PMC11528027; doi:10.1038/s41467-024-53253-x)
Supplement: Supplementary file 4 — Description of Additional Supplementary Files [file 41467_2024_53253_MOESM4_ESM.pdf]

Description of Additional Supplementary Files

Supplementary Data 1- Distribution of the variables included in the analysis by country

Supplementary Data 2- Bivariate associations between variables and MDR UTI, Profile regression (PR) variable selection probabilities
